# Evaluation of the performance of an in-house duplex PCR assay targeting the *IS6110* and *rpoB* genes for tuberculosis diagnosis in Cameroon

**DOI:** 10.1186/s12879-020-05523-4

**Published:** 2020-10-23

**Authors:** Henry Dilonga Meriki, Ndze Henry Wung, Kukwah Anthony Tufon, Nyeke James Tony, Irene Ane-Anyangwe, Fidelis Cho-Ngwa

**Affiliations:** 1 Tuberculosis Diagnostic Unit, Buea Regional Hospital, Buea, Cameroon; 2 Department of Microbiology and Parasitology, University of Buea, Buea, Cameroon; 3 Department of Biochemistry and Molecular Biology, University of Buea, Buea, Cameroon

**Keywords:** Sensitivity, In-house duplex PCR, Composite reference standard

## Abstract

**Background:**

Tuberculosis (TB) remains a major public health concern in many low-income countries accounting for approximately two-thirds of deaths in people living with human immunodeficiency virus (HIV) infection. With prompt, accurate and appropriate treatment, almost all TB disease can be cured. The present study was to evaluate the diagnostic performance of an in-house duplex PCR (D-PCR) using *IS1610* and *rpoB* specific primers in sputum samples from TB suspected patients.

**Methods:**

A hospital-based cross-sectional study was conducted at the Limbe and Buea Regional Hospitals of the South West Region of Cameroon from June 2016 to April 2017. Sputum samples, decontaminated with hypertonic saline/sodium hydroxide solution were centrifuged and pellets processed for smear microscopy, culture and DNA extraction. Suspected inhibition was resolved by serial dilution of genomic DNA. Results were compared to culture as gold standard as well as a Composite Reference Standard (CRS).

**Results:**

A total of 129 participants aged between 5 to 82 years were enrolled in to the study. The median age of the participants was 37 years (interquartile range, IQR: 27–50 years), with 54.3% being male. Forty-seven samples (36.4%) were positive by direct sputum microscopy, 49 (38%) by microscopy after concentration, 51 (39.5%) by culture and 62 (40.1%) by D-PCR. PCR inhibition was resolved in 85.7% (18/21) of the samples that had inhibition. The overall sensitivity, specificity, positive and negative predictive values, positive and negative likelihood ratios and area under the curve AUC) of the D-PCR was 93.5, 94, 94, 94%, 15.6, 0.005 and 89.0% respectively using CRS as reference. The sensitivities of D-PCR observed among different sample categories were 95.7, 87.5 and 87.5% for smear-and culture-positives, smear-negative/culture-positive, and clinically diagnosed cases respectively.

**Conclusion:**

*IS1610* and *rpoB* duplex PCR using relatively cheap decontamination and DNA extraction methods in addition to simple serial dilutions to resolve PCR inhibition shows high sensitivity in the diagnosis of paucibacillary tuberculosis.

## Background

Pulmonary tuberculosis (TB) is a leading cause of death worldwide from a single pathogen [1, 2]. Despite the availability of effective treatments, TB remains a major public health concern in many low-income countries [3]. With accurate and early diagnosis, coupled with correct treatment, almost all TB disease can be cured [4, 5]. The World Health Organization (WHO) has identified as one of its main pillars for the “End TB Strategy”, early diagnosis and systematic screening of contacts and high-risk groups [6]. Prompt and accurate diagnosis of active TB is required for rapid initiation of the right therapy to avoid the devastating effects of the late-stage disease as well as for public health intervention to reduce the risk of further spread in the community [7]. Despite increases in TB notifications in 2018, there is still a large gap of about 30% between the number of new cases reported and the estimated incident cases, due to a combination of underreporting of detected cases and under-diagnosis [8].

Although tuberculosis diagnosis in many countries particularly in low-income countries, is still reliant on older tools such as microscopy, new diagnostics are changing the landscape [9]. Motivated, partly, by the success and rollout of Xpert MTB/RIF, there is now considerable interest in new technologies with promising new tools particularly in molecular diagnostics. However, new diagnostics are yet to reach scale, and there are needs for greater convergence between diagnostics development and improvement in shorter-course tuberculosis drug regimens [9].

The most rapid method of identifying mycobacteria to the species level in clinical specimens is polymerase chain reaction (PCR) [10]. Theoretically, this test is capable of detecting a single *Mycobacterium tuberculosis* organism, its sensitivity is expected to be close to 100% [11]. Also, compared to Xpert MTB/RIF, in-house PCR can be customized from diverse sources of equipment and reagents in open markets that can render the procedure more affordable, feasible, and sustainable in technologically-challenged settings [12]. However, there is low sensitivity in PCR analysis of sputum attributed to the presence of inhibitors and low numbers of organisms [13]. Sample dilution, the addition of PCR facilitators, and the use of resin are some techniques that have been employed to improve on PCR amplification in the presence of inhibitors [11].

A previous study to determine the sensitivity of 5 specific primers namely *IS6110, IS1081, rpoB, oxyR* and *hupB* on culture *M. tuberculosis* isolates in Cameroon, demonstrate a 100% sensitivity for *IS6110* and *rpoB* genes when assayed in duplex PCR (D-PCR) [14]. As a follow up of these findings, this study was to evaluate the diagnostic accuracy of these two primers in sputum samples from clinical suspected patients using culture and Composite Reference Standard (CRS) as a gold standard to determine their suitability in routine diagnosis of pulmonary tuberculosis in Cameroon.

## Methods

### Study design and setting

This was a hospital-based cross-sectional study conducted between June 2016 and April 2017. Participants were enrolled from the Buea Regional Hospital (BRH) and the Limbe Regional Hospital (LRH) in the South West Region of Cameroon. A network of 238 TB diagnostic and treatment centres (DTCs) is functional throughout the 10 regions of Cameroon with 19 of them found in the South West Region. Buea and Limbe are among the major DTCs with high patient influx [15]. Tuberculosis is still an important public health concern in this region, where most people live in agro-industrial camps, characterized by overcrowding, a predisposing factor for TB [16].

### Study participants

The study enrolled patients aged ≥5 years including pregnant women presenting with signs and symptoms of TB, and who were able to produce good quality sputum (mucoid/purulent consistency and sample volume ≥ 2 mL). Excluded from the study were participants who refused to give their consent or whose parents/guardians refuse to give a proxy consent or assent and patients who presented with salivary specimens.

### Questionnaire administration

Data were collected from medical records of study participants and responses to a semi-structured questionnaire administered to obtain information on demographic data (age, sex), socioeconomic status (occupation, level of education, marital status) and behavioural patterns (smoking and alcohol intake). A clinical scoring system adapted from that of de Castro et al. [17] was generated to assess the association of clinical evidence to TB outcome. Each variable included in the scoring system was assigned a score when present with a weight ranging from 1 to 3. These variables include age ≤ 59 years (2), cough > 2 weeks (2), weight loss (1), fever (2), dyspnoea (1), fatigue (1), night sweats (3), chest pain (1), haemoptysis (1), contact with TB patients (1) and history of TB (1). These scores summed up to 16 and a cut-off score of **≥**9 was considered significant clinical evidence of TB.

### Sample collection, processing, culture and presumptive identification

Well-labelled sputum mugs were provided to participants presenting with a productive cough which has lasted for at least 2 weeks, and early morning sputum samples were collected following standard procedures. The patients were advised to deliver the sample to the laboratory on the same day of collection.

Smears were prepared for direct acid-fast staining (AFB) following standard microbiological procedures. Each sputum sample was digested/decontaminated using hypertonic saline/sodium hydroxide and modified Petroff method [18, 19]. Briefly, a total of 2 mL of 7% NaCl and 1.5 mL of 4% NaOH were added to every 2 mL of sputum sample contained in a 50 mL conical tube, vortexed and incubated at 37 °C for 20 min. After which, phosphate buffer at pH 6.8 was added to the 50 mL mark and the tube centrifuged at 3000 g for 15 min at 10 °C. The processed sediments were used to inoculate Lowenstein Jensen (LJ) slants in duplicates, prepare smears on slides for microscopy and the remainder stored at − 20 °C for DNA extraction. The slants were incubated at 37 °C and examined weekly for eight consecutive weeks. A second smear was prepared for AFB after decontamination and centrifugation. All the smears were stained with Ziehl Neelsen stains following standard microbiological procedures.

Isolates were presumptively identified as members of the *Mycobacterium tuberculosis* complex (MTBC) based on colonial morphology on LJ medium and serpentine cording on Ziehl-Neelsen stained smears as previously reported [20]. Serpentine cording was defined as tight rope-like aggregates of acid-fast bacilli in which the long axis of the bacteria parallel the long axis of the cord. The uniform distribution or other arrangement was considered the absence of cording [20].

### DNA extraction and duplex PCR assay

The DNA extraction was performed by intermittent heating (95 °C) and freezing for 10 min each. The procedure was repeated thrice and the tubes were centrifuged at 15000 g for 7 min and 150 μL of the supernatant carefully transferred to a new sterile tube [21], 3 μL of which was used as a template for PCR amplification. Where suspected, inhibition of PCR was resolved by dilution of the template. The thermo-lysate was precipitated in absolute ethanol and pellets washed in cold 70% ethanol [22]. The pellets were re-suspended in 25 μL of sterile distilled water and used as stock for 10-fold serial dilution.

Genomic DNA was analyzed by D-PCR using previously published and specific primers [23, 24] for the identification of *IS6110* and *rpoB* genes (Table 1).

**Table 1:** Primers used and their sequences

Gene (PCR product size bp)	Primer name	Primer sequence (5′- 3′)	Annealing temperature
*rpoB* (235)	*rpoB*F	TACGGTCGGCGAGCTGATCCAAA	68 °C
	*rpoB*R	ACAGTCGGCGCTTGTGGGTCAAC	
*IS6110* (123)	*IS6110*F	CCTGCGAGCGTAGGCGTCGG	63 °C
	*IS6110*R	CTCGTCCAGCGCCGCTTCGG	

The primers were synthesized and purified commercially (SIGMA, Germany). The 25 μL PCR reaction mixture consisted of 12.5 μl of RedTaq PCR SuperMix (SIGMA, Germany), 3 μL of bacterial thermo-lysate, 1.5 μL each of the two flanking primers at a final concentration of 0.5 μM and 3.5 μL of PCR water. Positive and negative controls were also included in the assay. The positive control consisted of a DNA template from a culture with typical phenotypic characteristics of *Mycobacterium tuberculosis* complex (MTBC) and the negative control was made up of PCR water replacing template. Samples showing negative results were re-tested with serially diluted genomic DNA. The Peltier thermal Cycler was used for all the PCR amplifications.

The PCR cyclic conditions for the D-PCR were as follows; initial denaturation at 95 °C for 10 min, then 35 cycles at 94 °C for 1 min, 68 °C for 1 min, and 72 °C for 1 min, with a final elongation step at 72 °C for 10 min. The PCR products were analyzed by standard agarose gel electrophoresis on a 2% gel. The Direct Load wide-range DNA marker (Lot No. MKB22768V) (1 g/μL) was electrophoresed in parallel with the PCR products.

### Data management and analysis

Data analysis was carried out using Statistical Package for the Social Sciences version 22 (Chicago, Illinois) and the EPI info version 7.2.0.1. Counts, percentages, median and interquartile ranges were reported. Sensitivities, specificities, positive (PPV) and negative predictive values (NPV), negative (LR-) and positive (LR+) likelihood ratios and area under the curve (AUC) were calculated for the D-PCR compared to culture as well as CRS (a combination of direct AFB and concentrated AFB microscopy, culture and clinical evidence) as references. Chi-square comparison was done at significance level of 0.05.

### Ethics statement

The study was approved by the University of Buea Institutional Review Board (No. 2016-05-0523). Administrative approvals were obtained from the Regional Delegation of Public Health, Buea and the study host institutions. Written informed consent was obtained from participants ≥21 years. Written assent was obtained from parents/guardians of partcipants < 21 years in addittion to a proxy consent from these participants.

## Results

### Characteristics of the study population

A total of 129 participants were enrolled for the study; comprising 59 (45.7%) females and 70 (54.3%) males with a median age of 37 years (IQR: 27–50 years). Ninety-seven participants were enrolled from the BRH and 32 from the LRH. The majority (47.3%) of the participants were between the ages of 20–39 years, while 9.0 and 14.0% were teenagers and adults older than 60 years respectively. Close to 40% of the participants were unemployed (36.5%) and 58.3% had attained at least a secondary level of education (> 11 years of formal education). Majority of the participants did not smoke, although about half of them confirmed taking alcohol (49.6%). Forty-seven per cent of the participants had a TB symptoms clinical score of at least 9 (Table 2).

**Table 2:** Socio-demographic, behavioural characteristics and clinical history of the study population

Variables	N	Category	n (%)
**Gender**	**129**	Female	59 (45.7)
		Male	70 (54.3)
**Age group (years)**	**129**	0–19	12 (9.0)
		20–39	61 (47.3)
		40–59	38 (29.5)
		≥ 60	18 (14.0)
**Employment status**	**126**	Unemployed	46 (36.5)
		Self-employed	49 (38.9)
		Employed	31 (24.0)
**Years of formal education**	**127**	< 11 years	53 (41.7)
		11–14 years	41 (32.3)
		> 14 years	33 (26.0)
**Alcohol intake**	**127**	No	64 (50.4)
		Yes	63 (49.6)
**Smoking**	**129**	No	121 (93.8)
		Yes	8 (6.2)
**TB symptoms clinical score**	**129**	< 9	68 (52.7)
		≥ 9	61 (47.3)
**HIV Status**	**129**	Positive	55 (42.6)
		Negative	46 (35.7)
		Unknown	28 (21.7)
**Previous TB disease**	**129**	Yes	27 (20.9)
		No	94 (72.9)
		Unknown	8 (6.2)

### Clinical presentation of study participants at enrolment

**Fig. 1:**
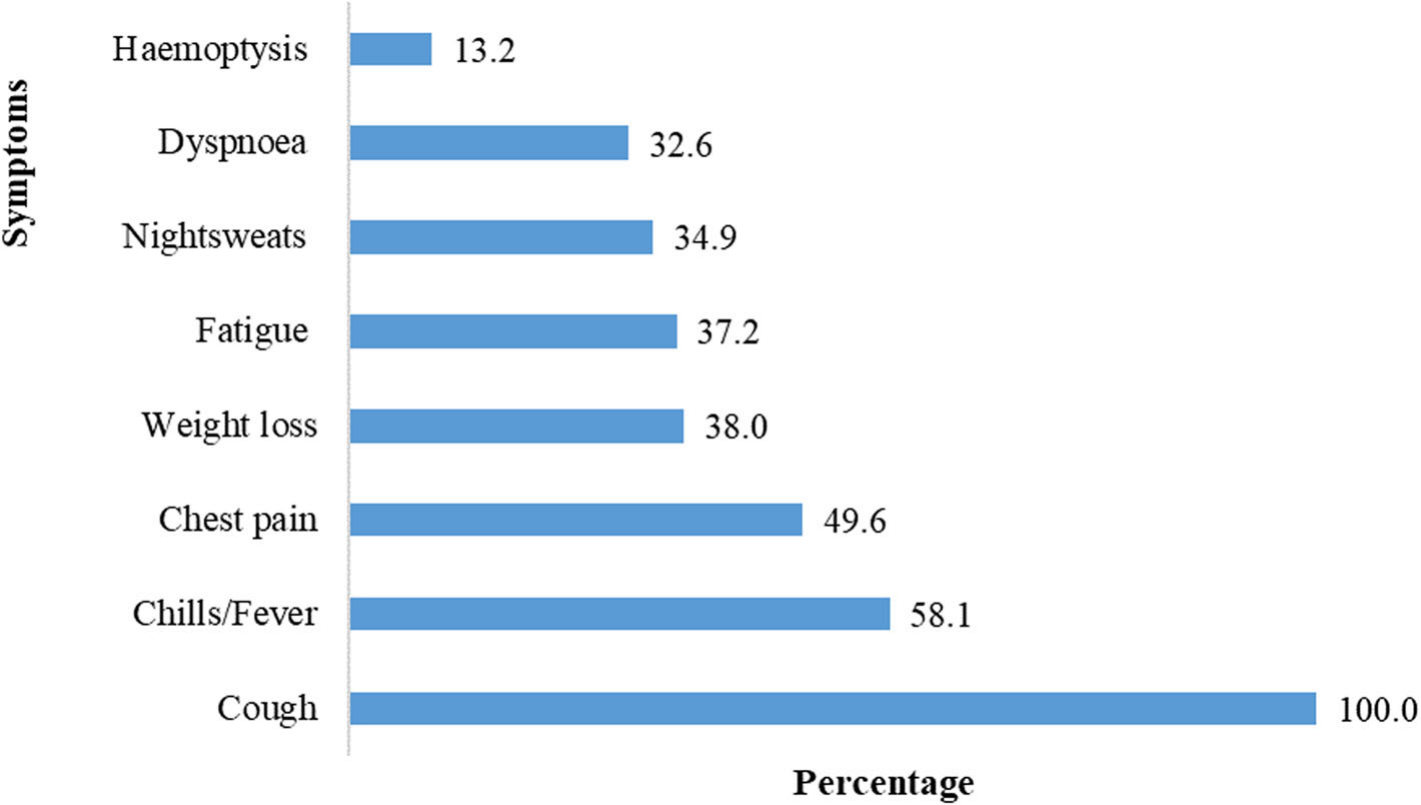
Clinical presentation of study participants at enrolment

During the selection of study participants, 58.1% of them complained of having fever/ chills, 38.0% had lost weight, 49.6% had chest pain, 32.6% had difficulty in breathing, 37.2% complained of fatigue, 13.2% were coughing with blood and 34.9% had night sweats (Fig. 1).

### Prevalence of tuberculosis in the study population

**Fig. 2:**
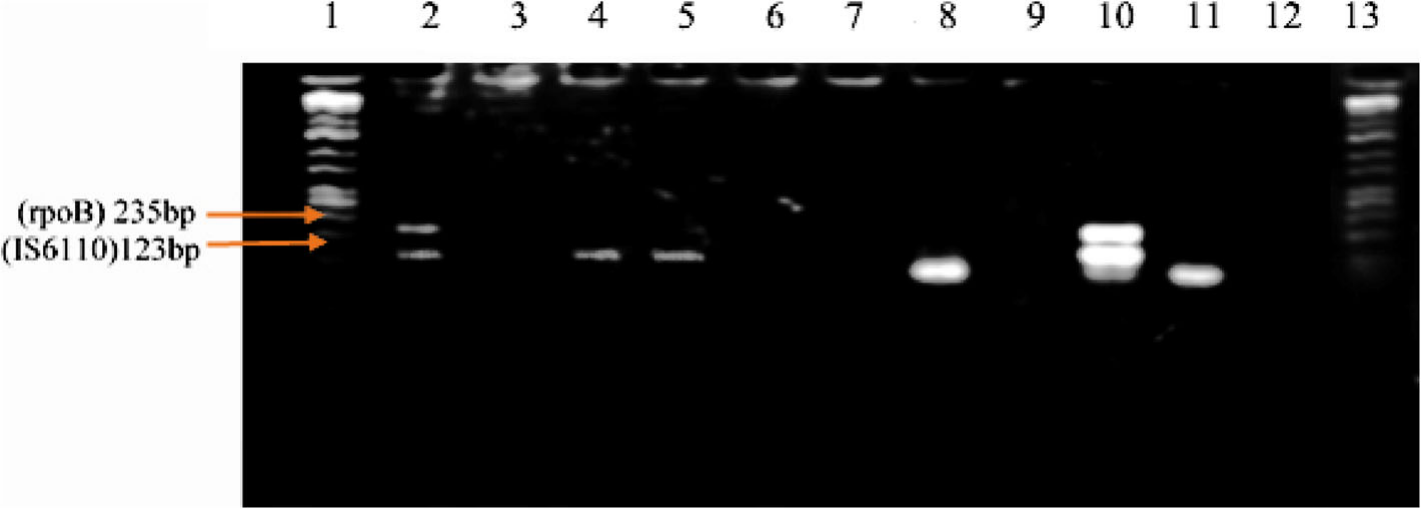
Sample PCR results. [Lane 1 and 13: MWM, Lane 3: Negative control; Lane 10: Positive control; Lanes 6, 7, 9, and 12: Negative PCR; Lane 4 and 5 (faint bands), lanes 8 and 11: positive samples for *IS6110* only; Lane 2: Positives for both IS6110 and *rpoB*]

Of the 129 study participants, direct microscopy identified 47 cases (36.4%), while 49 cases (38.0%) were identified by microscopy after concentration and 51 (39.5%) by culture. Meanwhile, PCR identified 57 cases (44.2%) for the *rpoB* gene, 61 cases (47.3%) for the *IS6110* locus and 62 (48.1%) by duplex-PCR (Fig. 2).

**Fig. 3:**
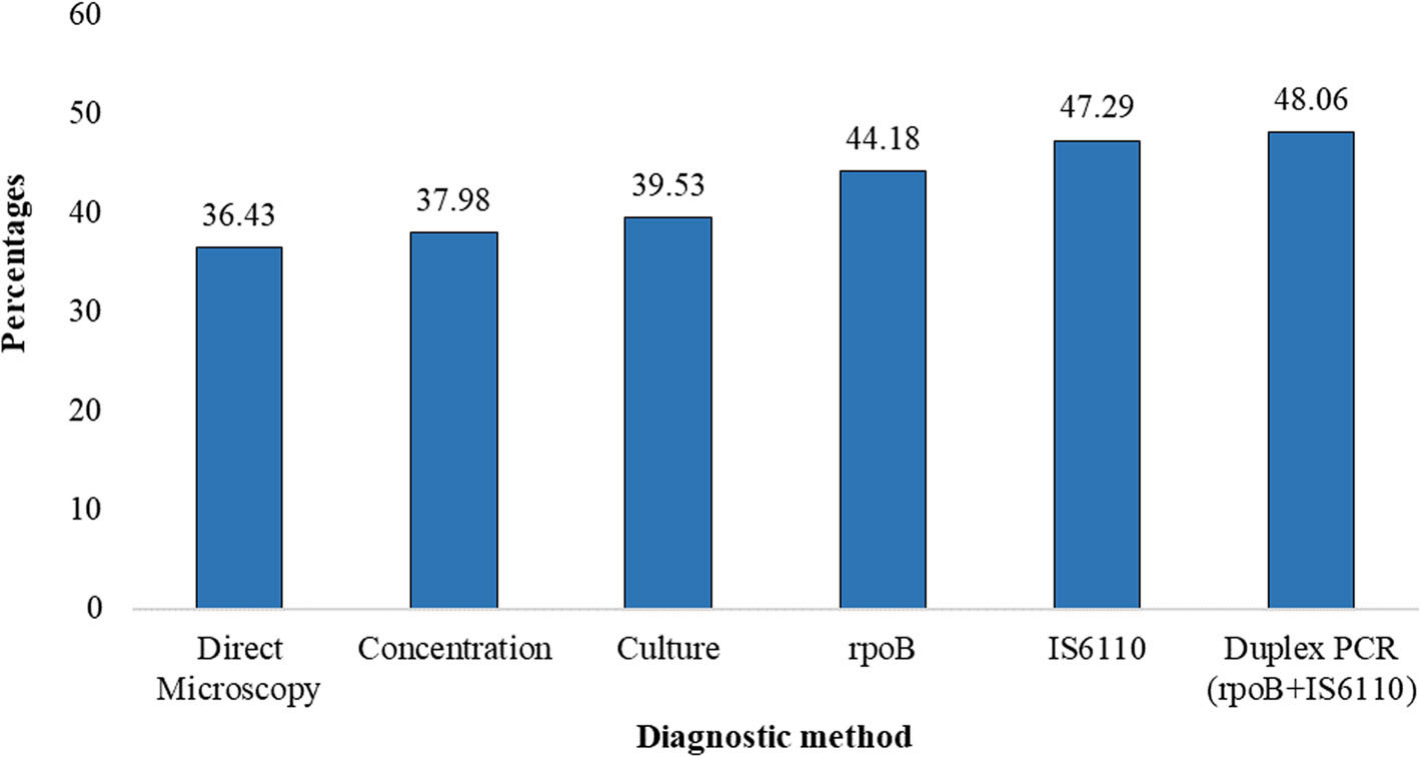
Variation in tuberculosis detection rate by the different diagnostic techniques

Overall, 65 (50.4%) samples were positive by direct microscopy, microscopy after concentration, culture and D-PCR combined. Figure 3 shows the variation in the detection rate of the different diagnostic methods.

### Resolution of PCR amplification inhibitors

**Fig. 4:**
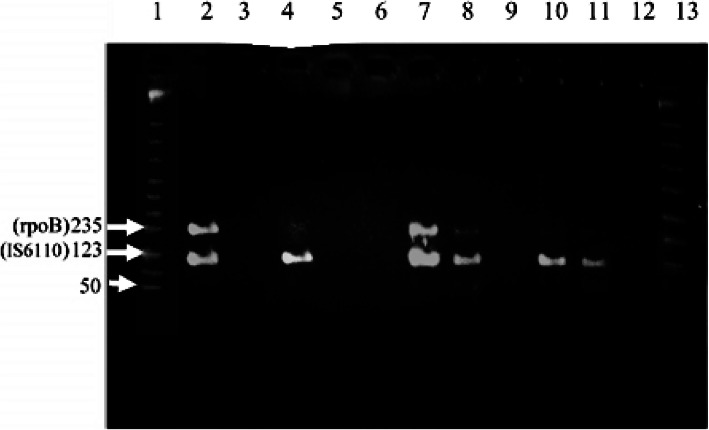
PCR Results after decimal dilution of samples. [Lane 1 and 13: MWM; Lane 2: Positive control; Lane 3: Negative control; Lane 4: Positive sample for IS6110 (10^− 2^); Lanes 7 (10^− 1^), Lane 8, 10, and 11: Positive for D-PCR; Lanes 5, 6, 9 and 12: Negative samples]

A total 21 samples could not be amplified after the first PCR runs although they were positive for either culture and/or microscopy (12 samples with bacterial load rated at AFB 3+, 1 AFB 2+, 1 AFB 1+ and 6 AFB negative but culture-positive). However, after a 10-fold serial dilution, inhibition was resolved in 18 (85.7%) of these samples. From the results of microscopy, samples that were at 0 (negative), or 1+ positive level, required less than 100 fold or no dilution, while samples ≥2+ positive level required 100-fold dilution. The results of D-PCR after serial dilution are shown in Fig. 4.

### Comparison of duplex-PCR and different result combination of smear and culture methods

Six cultures were contaminated, one of which was positive by both microscopy and D-PCR. Meanwhile, 7 of the 8 smear-negative and culture-positive cases were confirmed positive by D-PCR. Equally, of the 8 participants placed on treatment based on clinical evidence alone, 7 were confirmed positive by D-PCR. Table 3 shows the PCR results of the different sample categories.

**Table 3:** Comparison of Duplex-PCR and different result combination of other diagnostic methods tested

Sample category			PCR Results					
	n	%	***IS6110***		***rpoB***		D-PCR	
			(−)	(+)	(−)	(+)	(−)	(+)
Smear (+) Culture (+)	44	34	3	41	4	40	2	42
Smear (−) Culture (+)	7	5.4	1	6	3	4	1	6
Smear (−) Culture (−)	62	48	58	4	58	4	58	4
Smear (+) Culture contaminated	1	0.7	0	1	0	1	0	1
Smear (−) Culture contaminated	5	3.9	5	0	5	0	5	0
Smear (+) Culture (−)	2	1.6	0	2	0	2	0	2
Clinically diagnosed TB	8	6.2	1	7	2	6	1	7
Total	129	100	68	61	72	57	67	62

(−) = negative, (+) = Positive

**Fig. 5:**
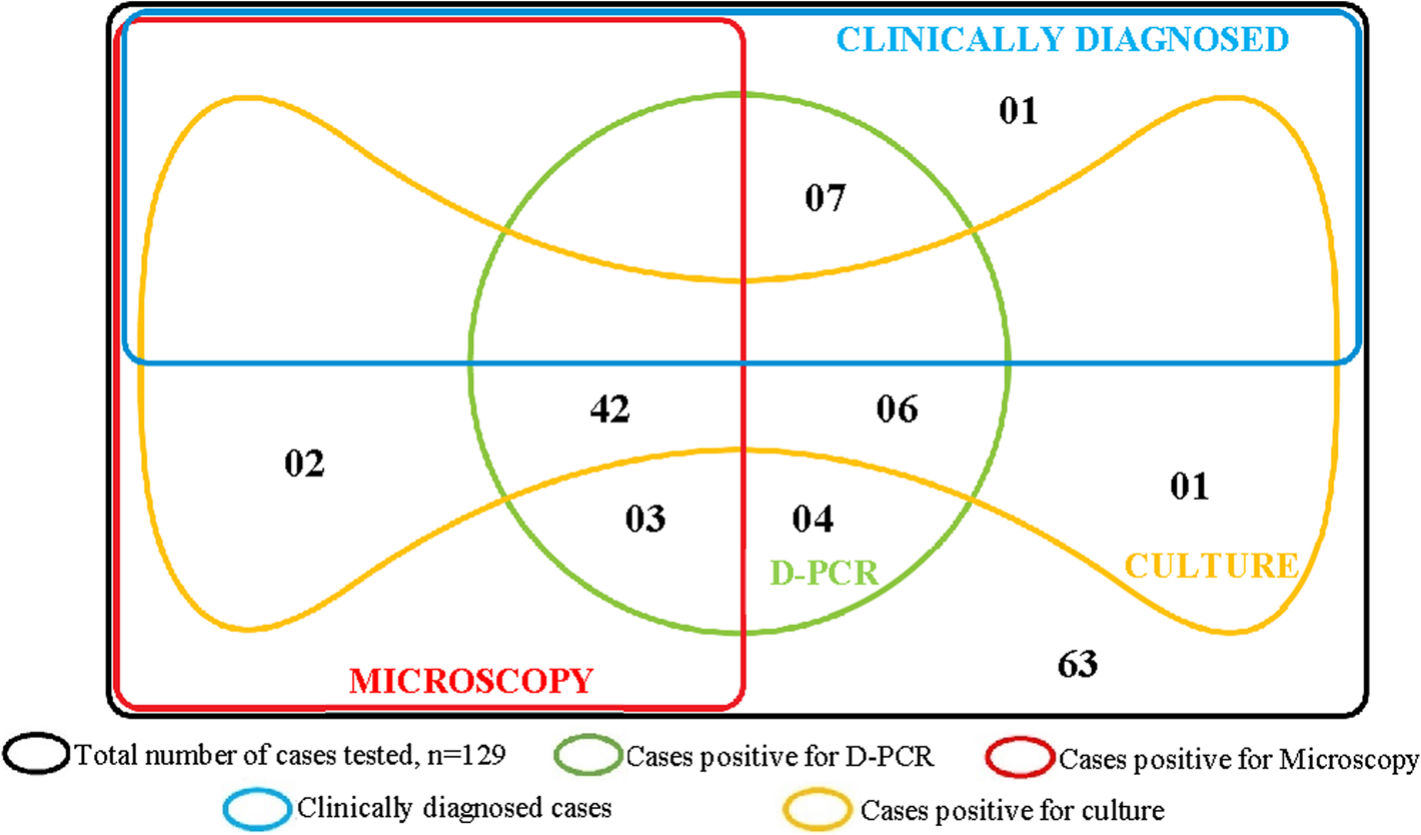
Venn diagram showing the synopsis detection level of the various diagnostic methods

As illustrated by the Venn diagram in Fig. 5, D-PCR (Green) detected TB in all except 4 cases detected by other methods. D-PCR also detected up to 87.5% (7/8) of clinically diagnosed cases missed by these routine diagnostic methods. Cases that could not be diagnosed by culture due to contamination were detected by D-PCR indicating that sputum contamination does not affect D-PCR outcome.

### Measures of diagnostic accuracy compared to composite reference (CRS) and culture as gold standards

When analysed against CRS as the gold standard, the sensitivities of direct microscopy, microscopy after concentration, LJ culture and D-PCR were 75.8, 79, 83.6, and 93.5% respectively (Table 4).

**Table 4:** Values of diagnostic accuracy with CRS as standard

	Diagnostic Methods					
Parameters	Direct microscopy	Concentrated microscopy	LJ Culture ***n*** = 123^a^	***rpoB***	***IS6110***	D-PCR
No. of samples positive	47	49	51	57	61	62
No. of samples negative	82	80	72	72	68	67
**Measures of diagnostic accuracy**						
Sensitivity (%)	75.8	79.0	83.6	87.1	91.9	93.5
Specificity (%)	100	100	100	94.0	94.0	94.0
PPV	1	1	1	0.93	0.93	0.94
NPV	0.82	0.84	0.86	0.89	0.93	0.94
LR+	∞	∞	∞	14.5	15.3	15.6
LR-	0.242	0.21	0.16	0.14	0.02	0.005

PPV (positive predictive value); NPV (negative predictive value); LR+ (positive likelihood ratio); LR- (negative likelihood ratio). ^a^ excluding contaminated cultures

However, when the culture was used as a reference, the sensitivity values increased, while there was a general drop in specificity values for all detection methods compared to values obtained with CRS as reference. The LR+ also dropped to the extent that PCR was moderately useful as a diagnostic test (LR+ < 10), though, D-PCR was good at “ruling out” TB disease (LR- <  0.1) (Table 5).

**Table 5:** Measures of diagnostic accuracy using culture as the reference standard

	Diagnostic method				
Parameters	Direct microscopy	Concentrated microscopy	***rpoB***	***IS6110***	D-PCR
Number of samples negative	77	75	66	63	62
Number of samples positive	46	48	57	60	61
**Measures of diagnostic accuracy**					
Sensitivity (%)	86.3	90.2	88.2	92.2	94.1
Specificity (%)	97.2	97.2	83.3	81.9	81.9
PPV	0.96	0.96	0.79	0.78	0.79
NPV	0.91	0.93	0.91	0.94	0.95
LR+	30.8	32.2	5.3	5.1	5.2
LR-	0.14	0.1	0.14	0.1	0.07

PPV (positive predictive value); NPV (negative predictive value); LR+ (positive likelihood ratio); LR- (negative likelihood ratio)

The sensitivity of the D-PCR in the smear (+)/culture (+) category was 95.7% and dropped to 87.5% in both paucibacillary smear-negative/culture-positive and clinically diagnosed TB categories, as shown in Table 6.

**Table 6:** Sensitivity of Duplex PCR in different sample categories with CRS as reference

Category	n	D-PCR results		Sensitivity (%)
		Negative	Positive	
Smear (+) Culture (+)	47	2	45	95.7
Smear (−) Culture (+)	8	1	7	87.5
Clinically Diagnosed TB	8	1	7	87.5
Total	63	4	59	93.5

Overall, D-PCR detected significantly more TB cases (*p* <  0.001): 17, (20.7%), 15 (18.8%) and 13 (18.1%) when compared to direct microscopy; concentrated microscopy and LJ cultures respectively (Table 7).

**Table 7:** Comparison of D-PCR and routine diagnostic methods

Diagnostic methods	Outcome		D-PCR, n (%)		Chi-square	***p***-value
		n	Positive	Negative		
Direct microscopy	Positive	47	45 (95.7)	2 (4.3)	67.3	< 0.001
	Negative	82	17 (20.7)	65 (79.3)		
Concentrated microscopy	Positive	49	47 (95.9)	2 (4.1)	72.5	< 0.001
	Negative	80	15 (18.8)	65 (81.3)		
LJ cultures	Positive	51	48 (94.1)	3 (5.9)	69.1	< 0.001
	Negative	72	13 (18.1)	59 (81.9)		

**Fig. 6:**
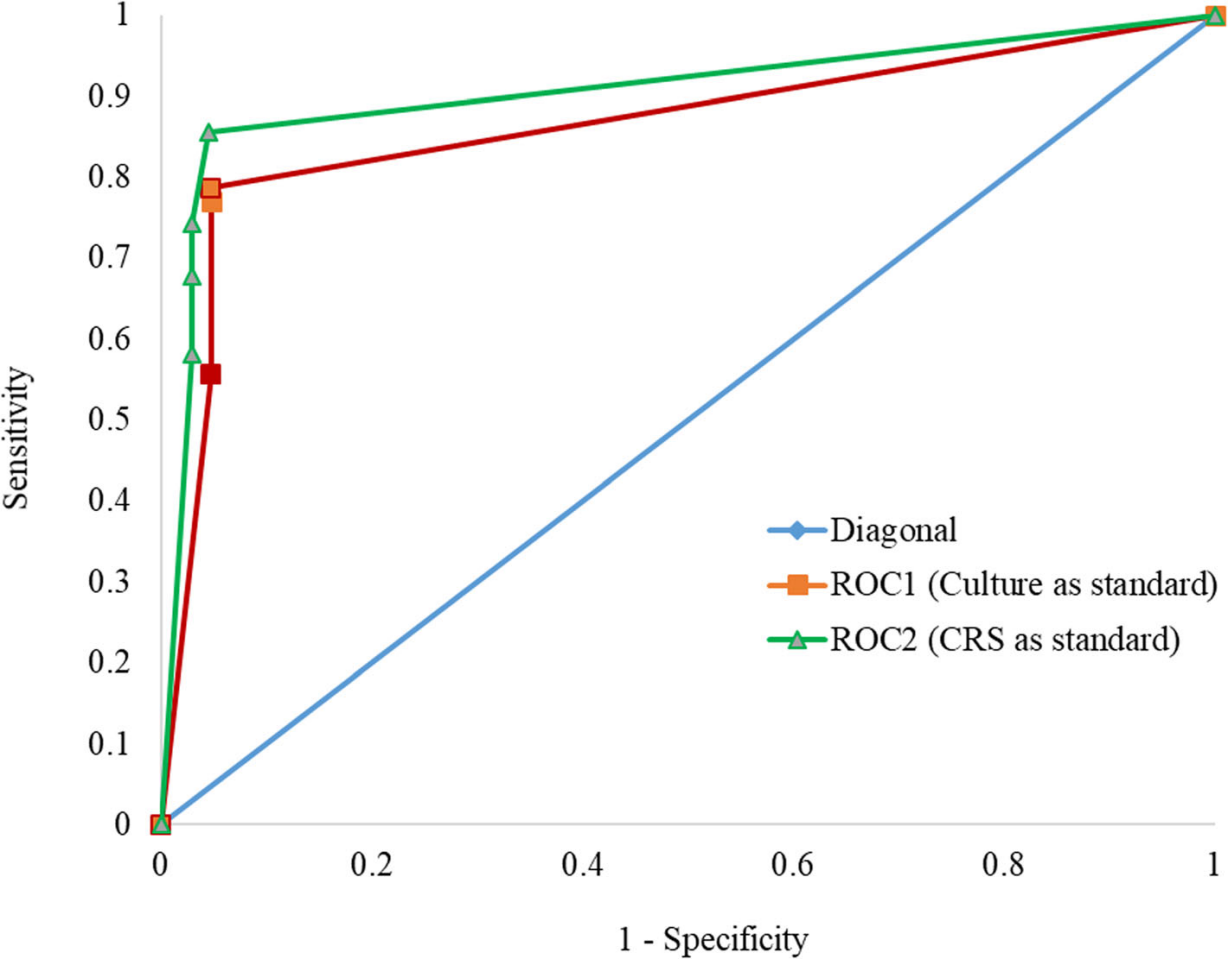
Receiver operating curve (ROC) of Duplex PCR with Lowenstein Jensen culture (ROC 1) and Composite reference standard (ROC 2)

The Area under the curve (AUC) was 0.864 (CI: 0.793–0.934, p < 0.001) using culture as standard for the D-PCR (ROC1) and 0.890 (CI: 0.827–0.954, *p* < 0.001) using composite reference (ROC2) (Fig. 6). Both standards showed that the D-PCR is a very good diagnostic test (0.8 < AUC < 0.9).

## Discussion

It has been estimated that a rapid TB diagnostic test with at least 85% pooled sensitivity for smear-positive and smear-negative cases and 97% specificity could save approximately 400,000 lives annually [25]. This study determined the diagnostic accuracy of D-PCR using primers (*IS6110* and *rpoB*) in direct sputum samples, hitherto established to be sensitive and specific [14].

The D-PCR performance was better compared to GeneXpert and another Multiplex PCR (*IS6110 + MPT64 + Protein antigen b*) carried out in South Africa [26]. Sensitivities of 83.8% for GeneXpert and 87.6% for Multiplex assay were reported, while our PCR format had a sensitivity of 93.5%. Meanwhile, specificity, PPV and NPV reported for GeneXpert were 70.4, 91.7, and 52.8% and 88.9, 96.8 and 56.6% for the multiplex format respectively. Compared to our D-PCR format, a specificity of 94%, PPV of 94% and NPV of 94% was obtained. However, this South African study [26] used *Mycobactertium* growth indicator tubes (MGIT) as a reference. Also, Gopinath and Singh in 2009, developed a triplex PCR assay targeting *hsp65* (genus-specific), *cfp10* (*Mycobacterium tuberculosis complex* specific) and 16S – 32S Internal Transcribed Region (*Mycobacterium avium* specific) to detect and simultaneously differentiate in a single tube *Mycobacterium tuberculosis*, *M. avium* and other species of *Mycobacteria.* They reported a sensitivity of 97% that is higher than that in our study, although the specificity (94.9%) is similar. However, their study involved both pulmonary and extrapulmonary samples [27].

Among the different sample categories, the performance of the D-PCR was in accord with a similar study carried out in India [28]. The format that targeted *mpb64 + IS6110* reported a sensitivity of 96% in bacteriologically confirmed cases and 88.8% in clinically suspects cases. Using the same CRS, this study obtained results similar to our study, where we obtained a sensitivity of 95.7 and 87.5% respectively in bacteriologically confirmed and clinically suspected cases. However, their overall sensitivity (90.5%) was lower than ours (93.5%), given that all our samples were sputa that has been reported to be associated with higher PCR inhibition rate [28]. Raj et al. in 2016 reported a PPV, NPV, and an accuracy of 100, 83, and 93% respectively, showing a better degree of accuracy and PPV compared to our study [PPV = 94%, and AUC = 89.0% (CI: 82.7–95.4)].

Four false-positive PCR results were recorded; the samples were from cases with a history of contact (3/4) and/or history of past TB disease (3/4) one of whom was a defaulter and one completed treatment less than a year ago. According to a previous study, PCR can remain positive for more than 1 year after the initiation of anti-tuberculous treatment [29]. It is highly probable that the false positives might be relapsed cases that were missed by the conventional diagnostic methods or cases harbouring non-viable bacilli.

Furthermore, four false-negative PCR results were recorded; two smear-positive/culture-positive, one culture-positive/smear-negative and one clinically diagnosed TB case. False negativity in nucleic acid amplification (NAA) tests for sputum has always been linked to the presence of inhibitors (including high amounts of genomic DNA), and a low number of bacilli [13, 14]. However, because only presumptive identification of isolates (no speciation) was done before PCR, the possibility of detecting species other than *Mycobacterium tuberculosis* complex cannot be ruled out.

Although procedures abound on how to circumvent the problems of inhibition, most of the techniques are expensive or laborious and may either lead to loss of DNA pellets, the introduction of exogenous inhibitors and or damage to template [14]. The choice of sample dilution in this study was appealing because it is cheap, fast and easy to perform and in the end, suspected inhibition and /or low copy number was resolved in 85.7% (18/21) of samples subjected to this method. A study carried out by Dӧşkaya et al. [30] in the diagnosis of *Pneumocystis jirovecii* reported an initial PCR inhibition rate of 26.3% that was completely resolved by two-fold serial dilution. Nevertheless, they used both sputum and extra-pulmonary samples, extraction kits, and dilutions were performed on direct samples rather than genomic DNA as in our study.

The use of simple boiling can yield a good amount of impure DNA when fragile cells are involved, but with rigid cells like Mycobacteria and yeast, freeze-thawing treatment is more injurious to the membrane than heating alone [21]. In another study, template DNA from 30 samples obtained by the standard Cetyltrimethylammonium bromide (CTAB) method and by simple boiling were subjected to PCR. Thermo-lysate gave 100% positive rate while the standard protocol gave 83.3% positive rate [14]. These results show an advantage of the boiling method over standard protocol and freeze-heating method used in this study, over simple heating.

That notwithstanding, there are some limitations to this study. Firstly, liquid culture (mycobacterial growth indicator tubes) was not used in conjunction with LJ medium which could have improved the sensitivity of culture methods. Secondly, the isolates used in this study were only presumptively identified as *M. tuberculosis* complex strains morphologically. Therefore, false positives and/or cross-reactivity with other *Mycobacterium* spp. could not be determined implying in clinical practice the detection of *Mycobacteria species* other than MTB complex using this assay cannot be ruled out. Sequencing of the PCR products could have mitigated these effects but was not perform at the time of the study, so, results should be interpreted with this in mind. Finally, although the focus of this study was to evaluate the diagnostic sensitivity of the assay, analytical sensitivity was equally important but was not done, hence results should be interpreted with caution.

## Conclusions

This study demonstrates that duplex PCR, targeting *IS6110* and *rpoB* genes can improve diagnosis in smear-negative and culture-negative paucibacillary tuberculosis specimens, which pose significant diagnostic challenges in routine clinical practice in resource-constraint settings like Cameroon.

## Data Availability

The dataset supporting the conclusions of this article is included in the article.

## Abbreviations

AFB: Acid-fast baccilli
AUC: Area under the curve
CRS: Composite reference standard
D-PCR: Duplex polymerase chain reaction
IS6110: Insertion sequence 6110
LJ: Lowenstein Jensen medium
LR-: Negative likelihood ratio
LR+: Positive likelihood ratio
MTBC: *Mycobacterium tuberculosis* complex
NAA: Nucleic acid amplification
NPV: Negative predictive value
PP: Positive predictive value
ROC: Receiver operating curve
rpoB: RNA polymerase B
